# Health-related quality of life in IBD patients with long COVID under China’s normalized COVID-19 management

**DOI:** 10.1186/s12879-026-14323-1

**Published:** 2026-09-01

**Authors:** Runnan Wang, Lei Lei, Peizhao Liu, Mengxuan Li, Lin Wu, Juanhan Liu, Haiyang Jiang, Jianan Ren, Chaogang Fan, Tao Zheng

**Affiliations:** 1 Department of General Surgery, BenQ Medical Center, The Affiliated BenQ Hospital of Nanjing Medical University, Nanjing, China; 2 Department of General Surgery, 72nd Group Army Hospital, Huzhou, Zhejiang China; 3 Research Institute of General Surgery, Affiliated Jinling Hospital, Medical School, Nanjing University, Nanjing, China; 4 Department of Abdominal Acute Care Surgery, General Surgery Center, Shanghai General Hospital, Shanghai Jiao Tong University School of Medicine, Shanghai, China

**Keywords:** Long COVID, Health-related quality of life, Inflammatory bowel disease, Normalized COVID-19 management

## Abstract

**Introduction:**

Although the COVID pandemic has gradually subsided, many patients have experienced complicated long-term symptoms, collectively known as “long-COVID”, which is commonly associated with persistent discomfort and decrease in quality of life. Individuals with immune-mediated inflammatory diseases, such as inflammatory bowel disease (IBD), are particularly vulnerable in the context of COVID infection, while the influence from “long-COVID” remains unrevealed. This cross-sectional study aims to assess health-related quality of life (HRQoL) among IBD patients with long COVID and to identify factors associated with HRQoL impairment.

**Methods:**

A cross-sectional survey was conducted from September 16 to October 10, 2023, among 235 IBD patients who had been treated at Nanjing BenQ Medical Center and Nanjing Jinling Hospital. The survey collected demographic data, COVID-19-related information, and HRQoL assessments using the Short Inflammatory Bowel Disease Questionnaire (SIBDQ). Statistical analyses, including Chi-square tests, non-parametric tests, and multivariate linear regression, were employed to identify factors associated with long COVID and HRQoL.

**Results:**

Among 235 participants, 221 (94.04%) reported COVID-19 infection and 15 of 221 (6.79%) met the study definition of long COVID. Vaccination status differed by long-COVID status in an exploratory unadjusted comparison (*p* = 0.033); this finding was based on only 15 cases and was not adjusted for confounding. Participants with long COVID had a lower median SIBDQ score than those without long COVID (43.00 vs. 53.00; *p* = 0.009). In the multivariable model, any post-COVID-19 symptoms and symptom duration were entered as nested indicators. A duration of ≥ 4 weeks was associated with an additional 8.52-point lower SIBDQ score beyond the association of any post-COVID-19 symptoms (B=-8.52; 95% CI, -13.85 to -3.18; *p* = 0.002).

**Conclusion:**

Participants meeting the study definition of long COVID reported lower HRQoL than those without long COVID. However, because validated IBD activity measures were not collected, the observed difference cannot be separated from the possible effect of underlying IBD activity. The vaccination finding is exploratory. Larger prospective studies with standardized disease-activity assessment are required to confirm these associations.

**Supplementary Information:**

The online version contains supplementary material available at https://doi.org/10.1186/s12879-026-14323-1.

## Introduction

It has been four years since the outbreak of COVID-19 in Wuhan, Hubei Province, in December 2019. Despite the integration of COVID-19 into routine management, a substantial number of patients continue to experience persistent symptoms post-infection [1]. These symptoms often last for months, years, or longer, affecting multiple organ systems and overall well-being. Studies estimate that approximately 10% to 30% of COVID-19 patients suffer from post-COVID-19 symptoms [2, 3], commonly referred to as “long COVID,” also known as “acute post COVID-19 sequelae,” “post COVID-19 syndrome,” “post COVID-19 state,” or “post COVID-19 acute syndrome (PACS)“ [4, 5]. In this article, we use the term “long COVID”.

In 2021, the World Health Organization (WHO) defined long COVID as a condition occurring in individuals with a history of probable or confirmed SARS-CoV-2 infection, typically three months from the onset of COVID-19, with symptoms lasting at least two months and unexplainable by another diagnosis [2]. The U.S. Centers for Disease Control and Prevention (CDC) offer a slightly different definition, requiring symptoms to appear four weeks or more after the initial infection [4]. In this study, long COVID was defined as the presence of post-COVID-19 symptoms lasting more than four weeks after acute SARS-CoV-2 infection. This threshold was selected because our exploratory cross-sectional survey aimed to capture the broader burden of post-acute COVID-19 symptoms among patients with IBD, rather than to restrict the analysis to a narrower clinical case definition. In addition, published epidemiological studies have assessed post-acute COVID-19 symptoms or related health problems over varying follow-up windows, including 30–365 days after infection or the index encounter and 6–12 months after SARS-CoV-2 testing [1, 6].

Immune-mediated inflammatory diseases (IMIDs), such as inflammatory bowel disease (IBD), have drawn special attention during the pandemic due to the immunosuppressive therapies these patients often receive, increasing their infection risk [7–10]. Previous reviews have summarized that the COVID-19 pandemic substantially affected IBD clinical practice, including outpatient follow-up, endoscopic evaluation, treatment decision-making, telemedicine use, and patient access to care. In addition, early observational data from Northern Italy suggested that IBD-related factors, including disease activity, should be considered when evaluating COVID-19-related outcomes in this population [11, 12]. IBD, including Crohn’s disease (CD) and ulcerative colitis (UC), is a chronic inflammatory disorder of the gastrointestinal tract, usually treated with immunomodulatory or immunosuppressive drugs, which may further increase susceptibility to infections [8, 9, 13, 14]. Additionally, IBD patients may express high levels of angiotensin-converting enzyme 2 (ACE2) receptors, which serve as entry points for SARS-CoV-2, suggesting a potential interaction between COVID-19 and the intestinal pathways in IBD [7, 15]. Consistent with the gastrointestinal involvement of COVID-19, previous studies have reported small-bowel imaging abnormalities, mesenteric ischemic manifestations, and gastrointestinal endoscopic or histologic findings in patients with COVID-19 [16, 17]. These findings provide broader biological and clinical context for considering the potential relevance of COVID-19-related gastrointestinal manifestations in patients with pre-existing intestinal diseases such as IBD.

Long COVID has become a pressing public health concern. With the COVID-19 pandemic continuing for over four years, there is growing concern about the long-term effects of long COVID on physical, social, and emotional well-being, particularly its impact on Health-Related Quality of Life (HRQoL). This cross-sectional study aims to assess HRQoL among IBD patients with long COVID and to identify factors associated with HRQoL impairment.

## Methods

### Study design

This cross-sectional survey collected self-reported symptom data through web-based questionnaires from September 16 to October 10, 2023. The survey included demographic data, COVID-19-related information, long COVID symptoms (such as fatigue, brain fog, dizziness, gastrointestinal symptoms, and heart palpitations) [18], and HRQoL assessments (see Supplementary Material). Medication data were collected using a multi-select item covering 5-aminosalicylic acid (5-ASA), immunomodulators, biologics (infliximab, adalimumab, ustekinumab, vedolizumab), enteral nutrition, parenteral nutrition, and corticosteroids. Patients could select all applicable medications. Of note, formal disease activity assessment (e.g., CDAI, HBI, Mayo score, CRP, fecal calprotectin, or endoscopic evaluation) was not included in the questionnaire. As a remote web-based survey, our study prioritized accessibility and broad geographic reach; objective disease activity indices requiring in-person clinical or laboratory assessment were not feasible to collect under this design.

A total of 235 IBD patients, who had previously undergone treatment at hospitals include Nanjing BenQ Medical Center and Nanjing Jinling Hospital, completed our survey. Participants hailed from diverse regions and age groups to ensure the universality and representativeness of our findings. Patients’ privacy was rigorously protected, and no personal information was disclosed. Ethical approval was obtained from the Ethics Committee of Nanjing BenQ Medical Center (approval number: 2023-KL022). The study adhered to the Strengthening the Reporting of Observational Studies in Epidemiology (STROBE) guidelines.

### HRQoL assessment: Short Inflammatory Bowel Disease Questionnaire (SIBDQ)

In our study, the HRQoL of IBD patients was assessed using the SIBDQ. Compared to the more comprehensive Inflammatory Bowel Disease Questionnaire (IBDQ), the SIBDQ provides greater ease of completion and survey efficiency. This decision was made to improve patient compliance and reduce the burden on both respondents and researchers. Previous studies have demonstrated the high reliability and validity of both the SIBDQ and IBDQ as tools for measuring HRQoL in IBD patients [19, 20]. The SIBDQ consists of 10 questions divided into four domains: social, bowel, emotional, and systemic. Each question is rated on a 7-point Likert scale, with scores ranging from 1 (indicating severe problems) to 7 (indicating no problems). The total score can range from 10 to 70, with a score of 10 representing the poorest HRQoL and 70 indicating excellent HRQoL [21–23].

### Study endpoints

The primary endpoint was to evaluate HRQoL in IBD patients with long COVID, based on their SIBDQ scores, and explore the associated factors such as age, gender, IBD type, surgical history, and COVID-19 vaccination on long COVID development. The secondary endpoint focused on factors associated with HRQoL in IBD patients under normalized COVID-19 management.

### Exclusion and inclusion criteria

**Fig. 1:**
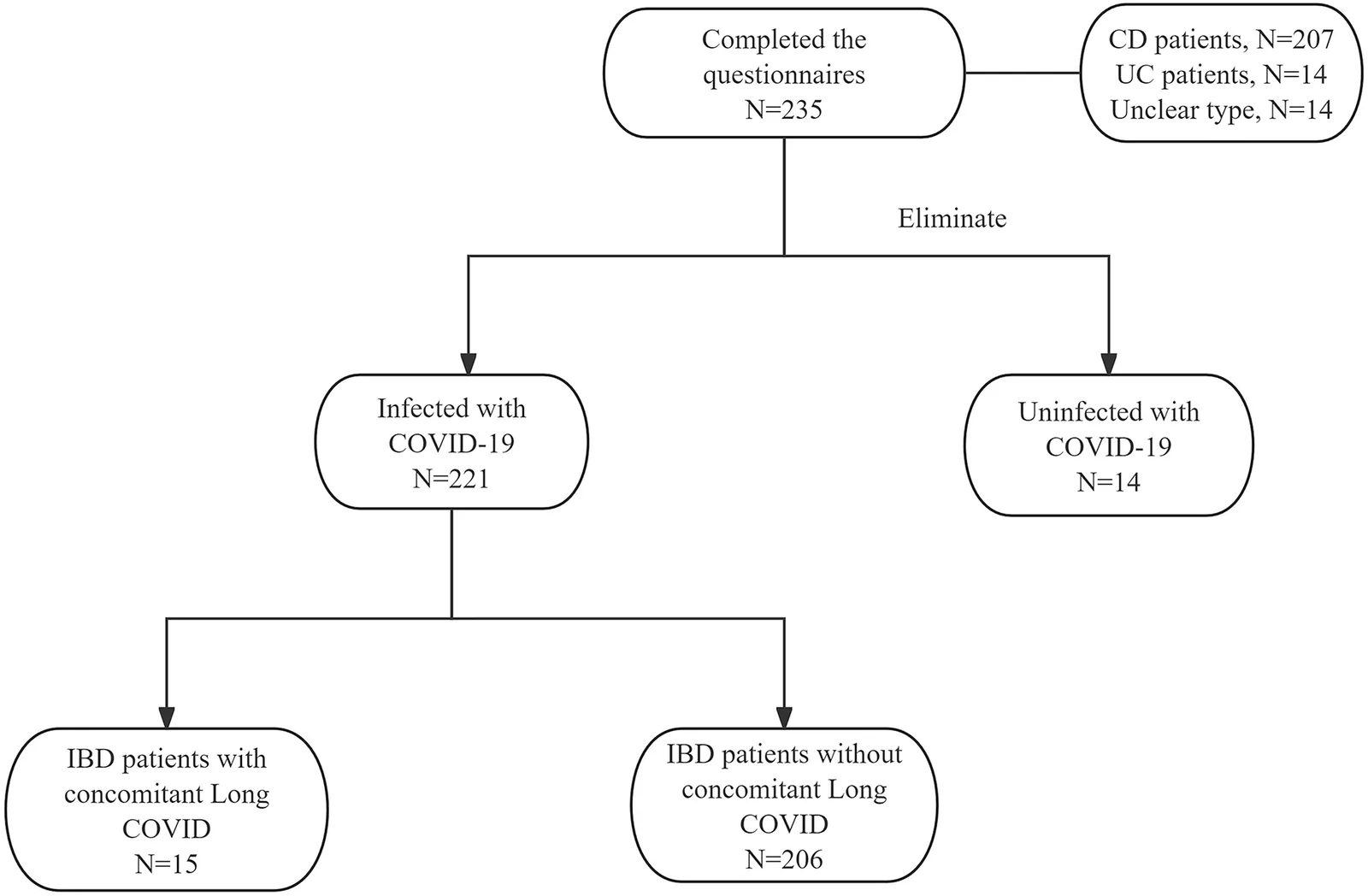
Patients flowchart

Patients who completed the questionnaire within the stipulated timeframe were included in the study. To improve the accuracy of the study, IBD patients who had not contracted COVID-19 were excluded (see Fig. 1). COVID-19 infection was defined as a positive nucleic acid test for the virus, while non-infection was defined as a negative nucleic acid test result.

### Statistical analyses

Statistical analyses were performed using SPSS version 26. Analyses of long COVID and HRQoL were restricted to the 221 participants with prior COVID-19 infection. Categorical variables were compared using Pearson’s chi-square test or a two-sided Fisher’s exact test, as appropriate. Continuous SIBDQ scores are presented as mean ± standard deviation when normally distributed and as median (Q1, Q3) otherwise; between-group comparisons used an independent-samples t-test for normally distributed variables and non-parametric tests for non-normally distributed variables. For comparisons involving three or more groups, ANOVA or the Kruskal–Wallis test was used, as appropriate.

In the descriptive and bivariate analyses, “any post-COVID-19 symptoms” and long COVID were analyzed separately as binary variables. “Any post-COVID-19 symptoms” compared participants reporting at least one self-reported symptom after acute infection, irrespective of duration, with those reporting no post-COVID-19 symptoms. Long COVID, defined as symptoms lasting ≥ 4 weeks, compared participants meeting this definition with all participants who did not meet the definition, including those without post-COVID-19 symptoms and those with symptoms lasting < 4 weeks.

Variables associated with SIBDQ scores in preliminary analyses (*p* < 0.05) were entered into a multivariable linear regression model with total SIBDQ score as the continuous outcome (see Table S2). In this regression model, the indicators for any post-COVID-19 symptoms and long COVID were entered simultaneously as nested binary variables. Under this coding, participants with no post-COVID-19 symptoms were coded as 0/0, those with symptoms lasting < 4 weeks as 1/0, and those with symptoms lasting ≥ 4 weeks as 1/1. Accordingly, the coefficient for any post-COVID-19 symptoms estimates the adjusted difference between participants with symptoms lasting < 4 weeks and those without post-COVID-19 symptoms, whereas the coefficient for long COVID estimates the additional adjusted difference between participants with symptoms lasting ≥ 4 weeks and those with symptoms lasting < 4 weeks. Unstandardized regression coefficients (B) with 95% confidence intervals are reported. A two-sided p-value < 0.05 was considered statistically significant.

## Results

### Baseline characteristics

A total of 235 IBD patients participated in the survey, among them, 131 (55.74%) individuals participated in our previous survey examining HRQoL among IBD patients after the end of the zero-COVID policy [24]. In this study, 131 (55.74%) were male and 104 (44.26%) were female. The majority (101, 42.98%) were aged between 17 and 40 years, which is also the age group most at risk for being diagnosed with IBD. CD was the predominant form of IBD, affecting 207 patients (88.09%), while UC was diagnosed in 14 patients (5.96%). Surgical intervention had been performed in 89 patients (37.87%). At the time of the survey, 126 patients (53.62%) were using 5-ASA as part of their treatment regimen. As the medication question allowed multiple selections, many patients reported combination therapy. Additionally, 57 patients (24.26%) had comorbidities such as hypertension, diabetes, kidney disease, etc. (see Table 1).

**Table 1:** Demographics of IBD patients (*N* = 235)

Variables	Value, *n*(%)
**Gender**	
Male	131 (55.74)
Female	104 (44.26)
**Age, years**	
≤ 16	65 (27.66)
17–40	101 (42.98)
>40	69 (29.36)
**Age at diagnosis of IBD, years**	
≤ 16	77 (32.77)
17–40	121 (51.49)
>40	37 (15.74)
**The highest level of education**	
Below high school	109 (46.38)
High school graduate	40 (17.02)
College/University	83 (35.32)
Graduate degree or above	3 (1.28)
**Type of IBD**	
CD	207 (88.09)
UC	14 (5.96)
Unclear type	14 (5.96)
**Operation for IBD**	
Yes	89 (37.87)
No	146 (62.13)
*Medications for IBD (Multiple selections allowed)	
No	18 (7.66)
Enteral nutrition	103 (43.83)
Parenteral nutrition	16 (6.81)
5-ASA (Mesalazine)	126 (53.62)
Glucocorticoid	5 (2.13)
Immunosuppressants (azathioprine, methotrexate)	19 (8.09)
Adalimumab	20 (8.51)
Infliximab	25 (10.64)
Ustekinumab	12 (5.11)
Vedolizumab	29 (12.34)
**Comorbidities**	
Yes	57 (24.26)
No	178 (75.74)

*For medication use, participants were allowed to select more than one treatment option; therefore, the percentages of medication categories are not mutually exclusive

### COVID-19 infection among IBD patients

Of the participants, 221 (94.04%) had contracted COVID-19, while 14 (5.96%) had not been infected. 92 (39.15%) had completed the full vaccination regimen (three or more doses), while 72 (30.64%) had received partial vaccination (one or two doses). Fever was the most common symptom following COVID-19 infection, affecting 186 patients (84.16%). After infection, the majority of IBD patients (145, 65.61%) opted for antipyretic medications to manage their symptoms. A total of 117 (52.94%) patients reported various post-COVID-19 symptoms, such as fatigue, memory loss, chest pain, and cough, with fatigue being the most prevalent, affecting 76 patients (34.39%) (see Table S1). The study also identified 15 (6.79%) IBD patients who experienced post-COVID-19 symptoms lasting longer than four weeks, meeting the criteria for long COVID as defined by the American CDC. In our study, 136 patients (61.54%) had contracted COVID-19 only once, while 47 (21.27%) had been reinfected within one month of their initial infection (see Table 2).

**Table 2:** COVID-19 related questions

Variables	Value, *n* (%)
**COVID-19 infection** No Yes **Vaccination** No Partial vaccination Full vaccination **Symptoms of COVID-19**	*N* = 235 14 (5.96) 221 (94.04) *N* = 235 71 (30.21) 72 (30.64) 92(39.15) *N* = 221
Fever Dry cough Fatigue Loss of smell Loss of taste Nasal congestion and runny nose Pharyngeal pain Conjunctivitis Muscle pain Diarrhea Asymptomatic **Drugs for COVID-19** Antipyretic drugs Hormone Antiviral Traditional Chinese medicines Baricitinib Tocilizumab None **Any post-COVID-19 symptoms** No Yes	186(84.16) 87(39.37) 130(58.82) 31(14.03) 28(12.67) 77(34.84) 110(49.77) 4(1.81) 64(28.96) 32(14.48) 12(5.43) *N* = 221 145(65.61) 5(2.26) 22(9.95) 41(18.55) 0 0 57(25.79) *N* = 221 104 (47.06) 117 (52.94)
**Long COVID**	*N* = 221
No	206 (93.21)
Yes	15 (6.79)
**Number of COVID-19 infections**	*N* = 221
Once	136 (61.54)
Twice	73 (33.03)
Three times	12 (5.43)
**Interval between COVID-19 reinfections**	*N* = 221
< 1 month	47 (21.27)
1–3 months	30 (13.57)
4–6 months	42 (19.00)
> 6 months	102 (46.15)

### SIBDQ scores

The mean SIBDQ score among IBD patients was 51.61. Social, emotional, systemic, and bowel function scores were 5.23, 5.00, 4.75, and 5.83, respectively.

### Factors associated with long COVID

In our study, vaccination status showed a significant association with the occurrence of long COVID (*p* = 0.033). Because only 15 long-COVID cases occurred and no adjusted model was fitted for this endpoint, this result should be considered hypothesis-generating and does not establish a protective effect of vaccination. No statistically significant differences were observed between long COVID occurrence and other factors such as gender, age, type of IBD, history of IBD surgery, or presence of comorbidities (*p* > 0.05) (see Table 3).

**Table 3:** Factors associated with long COVID

Variables	Long COVID (*n* = 15)	Non-Long- COVID (*n* = 206)	*P* value
**Gender**,** n (%)**			0.084
Male	5 (33.33)	116 (56.31)	
Female	10 (66.67)	90 (43.69)	
**Age**,** n (%)**			0.660
≤ 16 years	5 (33.33)	55 (26.70)	
17–40 years	5 (33.33)	92 (44.66)	
>40 years	5 (33.33)	59 (28.64)	
*Type of IBD,** n (%)**			0.457
CD	9 (60.00)	186 (90.29)	
UC	1 (6.67)	11 (5.34)	
**Operation for IBD**,** n (%)**			0.057
Yes	9 (60.00)	73 (35.44)	
No	6 (40.00)	133 (64.56)	
**Biological agents**,** n (%)**			0.869
Yes	5 (33.33)	73 (35.44)	
No	10 (66.67)	133 (64.56)	
**Comorbidities**,** n (%)**			0.531
Yes	2 (13.33)	51 (24.76)	
No	13 (86.67)	155 (75.24)	
**Drugs for COVID-19 infection**,** n (%)**			0.765
Yes	12 (80.00)	152 (73.79)	
No	3 (20.00)	54 (26.21)	
**COVID-19 vaccination**,** n (%)**			**0.033**
No and Partial vaccination	13 (86.67)	121 (58.74)	
Full vaccination	2 (13.33)	85 (41.26)	

* Participants with an unclear IBD type (*n* = 14) were excluded from the comparison of IBD type

### Factors associated with HRQoL

We compared SIBDQ scores across different groups. In our study, there was a significant difference in HRQoL between patients with and without long COVID, with those experiencing long COVID reporting lower HRQoL (*p* = 0.009; mean = 43.00 vs. 53.00). The HRQoL of IBD patients without comorbidities was better than that of patients with comorbidities (*p* = 0.002; mean = 54.00 vs. 49.00). Patients who developed symptoms after COVID-19 infection had lower HRQoL compared to asymptomatic patients (*p* < 0.001; mean = 49.00 vs. 57.00). Those who encountered difficulties in accessing medical care had significantly lower HRQoL than patients without such difficulties (*p* < 0.001; 48.36 ± 12.35 vs. 53.88 ± 8.05) (see Table 4).

**Table 4:** Factors associated with HRQoL

Variables	*n* (%)	SIBDQ scores	*P*
**Gender**,** M (Q₁**,** Q₃)**			0.343
Male	121 (54.75)	53.00 (44.00, 58.00)	
Female	100 (45.25)	53.50 (46.00, 61.00)	
**Age**,** years**,** M (Q₁**,** Q₃)**			0.233
≤16	60 (27.15)	52.00 (42.00, 57.00)	
17–40	97 (43.89)	54.00 (47.00, 60.00)	
> 40	64 (28.96)	52.50 (44.00, 60.00)	
**Type of IBD**,** M (Q₁**,** Q₃)**			0.084
CD	195 (88.24)	53.00 (46.00, 60.00)	
UC	12 (5.43)	47.00 (39.00, 53.75)	
Unclear	14 (6.33)	48.50 (43.50, 53.75)	
**Operation for IBD**,** M (Q₁**,** Q₃)**			0.180
Yes	82 (37.10)	51.00 (44.00, 58.00)	
No	139 (62.90)	54.00 (46.00, 60.00)	
**Comorbidities**,** M (Q₁**,** Q₃)**			**0.002**
Yes	52 (23.53)	49.00 (41.75, 54.00)	
No	169 (76.47)	54.00 (46.00, 61.00)	
**Vaccination**,** M (Q₁**,** Q₃)**			0.730
No	66 (29.86)	52.00 (43.00, 58.75)	
Partial vaccination	68 (30.77)	53.50 (44.75, 58.25)	
Full vaccination	87 (39.37)	53.00 (46.00, 60.00)	
**Duration of symptoms after COVID-19 infection**,** M (Q₁**,** Q₃)**			**0.009**
<4 weeks	206 (93.21)	53.00 (46.00, 60.00)	
≥ 4 weeks	15 (6.79)	43.00 (38.00, 55.00)	
**Number of COVID-19 infections**, **M (Q₁**,** Q₃)**			**0.033**
Once	136 (61.54)	54.00 (46.75, 61.00)	
Twice	73 (33.03)	51.00 (43.00, 57.00)	
Three times	12 (5.43)	49.50 (43.50, 53.50)	
**Any post-COVID-19 symptoms**,** M (Q₁**,** Q₃)**			**< 0.001**
Yes	117 (52.94)	49.00 (41.00, 55.00)	
No	104 (47.06)	57.00 (49.00, 62.00)	
*Difficulty in seeking medical care,** Mean ± SD**			**< 0.001**
No	130 (58.82)	53.88 ± 8.05	
Yes	91 (41.18)	48.36 ± 12.35	
**Susceptible to COVID-19**,** M (Q₁**,** Q₃)**			**< 0.001**
Yes	50 (22.62)	44.00 (36.00, 53.00)	
No	54 (24.43)	58.50 (54.00, 62.00)	
Not sure	117 (52.94)	52.00 (46.00, 57.00)	
**Unwilling to interaction**,** M (Q₁**,** Q₃)**			**< 0.001**
Yes	72 (32.58)	47.00 (42.00, 55.00)	
No	77 (34.84)	56.00 (51.00, 61.00)	
Not sure	72 (32.58)	53.00 (43.00, 60.00)	
**Affecting study or work**,** M (Q₁**,** Q₃)**			**< 0.001**
Yes	82 (37.10)	48.00 (42.25, 55.75)	
No	75 (33.94)	56.00 (50.00, 60.00)	
Not sure	64 (28.96)	54.00 (46.75, 61.00)	
**Affecting outdoor exercise**,** M (Q₁**,** Q₃)**			**< 0.001**
Yes	94 (42.53)	47.00 (42.00, 55.00)	
No	71 (32.13)	56.00 (51.50, 60.00)	
Not sure	56 (25.34)	54.50 (46.75, 61.25)	
**Using IBD medicine**,** M (Q₁**,** Q₃)**			0.274
Stop	21 (9.50)	48.00 (45.00, 60.00)	
Irregular	51 (23.08)	52.00 (44.00, 57.00)	
Regular	149 (67.42)	53.00 (46.00, 61.00)	

*SIBDQ scores for difficulty in seeking medical care were normally distributed and are presented as mean ± SD. The between-group comparison was performed using an independent-samples t-test. All other SIBDQ scores are presented as median (Q1, Q3) and were compared using non-parametric tests, as appropriate

Additionally, physical and psychological factors significantly associated with SIBDQ scores. IBD patients who believed they were more susceptible to COVID-19 had lower HRQoL compared to those who did not perceive themselves as vulnerable (*p* < 0.0001; mean = 44.00 vs. 58.50). Patients unwilling to engage in social interactions had lower HRQoL compared to those willing to socialize (*p* < 0.0001; mean = 47.00 vs. 56.00). IBD patients who reported a perceived negative effect of COVID-19 on their work or studies had significantly lower HRQoL scores than those who reported no such effect (*p* < 0.001; mean = 48.00 vs. 56.00). Similarly, lower HRQoL was observed among patients who reported COVID-19-related restrictions in outdoor activities compared with those who reported no such restrictions (*p* < 0.0001; mean = 47.00 vs. 56.00) (see Figure S1).

### Multivariate linear regression

In the multivariable linear regression, comorbidities were associated with a lower SIBDQ score (*p* = 0.004; B=-4.74; 95% CI, -7.91 to -1.57). The two COVID-19 symptom variables were nested rather than independent exposures. With duration included simultaneously, the coefficient for any post-COVID-19 symptoms compared participants with symptoms lasting < 4 weeks against those with no post-COVID-19 symptoms (*p* < 0.001; B=-7.68; 95% CI, -10.23 to -5.13). A duration of ≥ 4 weeks was associated with an additional 8.52-point decrement beyond any-symptom status (*p* = 0.002; B=-8.52; 95% CI, -13.85 to -3.18). Thus, B=-8.52 should be interpreted as the additional adjusted difference relative to symptoms lasting < 4 weeks, not as the total crude contrast between all participants with and without long COVID. Difficulty accessing medical care was also associated with a lower SIBDQ score (*p* < 0.001; B=-5.51; 95% CI, -8.20 to -2.82).

Regarding psychological factors, IBD patients who perceived themselves as more susceptible to COVID-19 had worse HRQoL compared to those who did not consider themselves more vulnerable (*p* < 0.001; B=-12.06; 95% CI, -15.74 to -8.39). Additionally, patients reluctant to engage in social interactions had significantly worse HRQoL compared to those who were willing to socialize (*p* < 0.001; B=-8.10; 95% CI, -11.27 to -4.93).

**Fig. 2:**
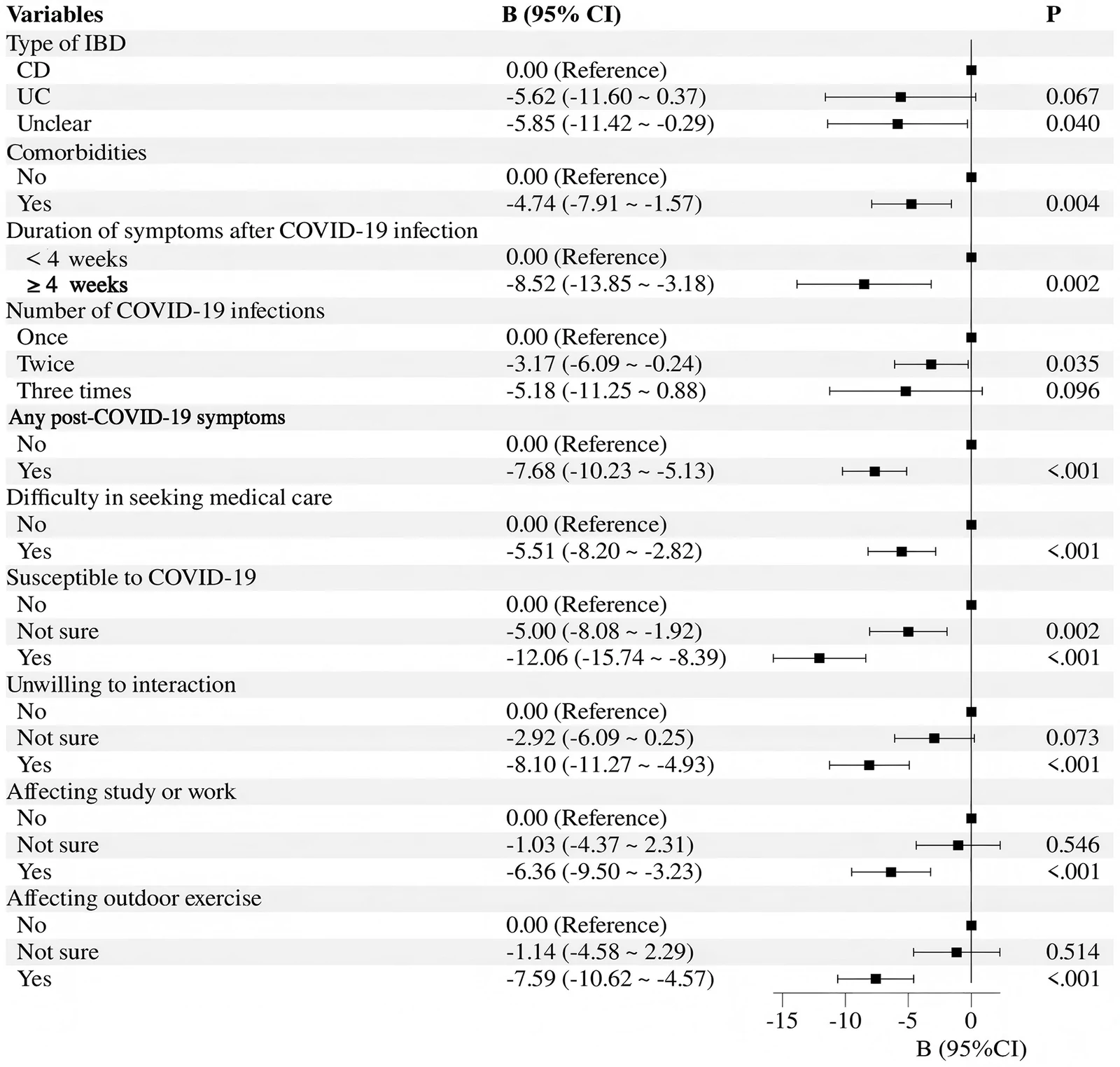
Multivariate linear regression

For physical factors, IBD patients who reported a perceived negative effect of COVID-19 on work or studies had lower HRQoL scores compared to those who were not affected (*p* < 0.001; B=-6.36; 95% CI, -9.50 to -3.23). Patients who reported a perceived negative effect of COVID-19 on their outdoor activities had lower HRQoL scores than those who reported no such effect (*p* < 0.001; B=-7.59; 95% CI, -10.62 to -4.57) (see Fig. 2).

## Discussion

We previously conducted a published study assessing COVID-19-related HRQoL among IBD patients following the end of China’s zero-COVID policy [24]. However, our earlier research did not address the issue of long COVID in IBD patients. Therefore, the current study serves as a continuation of our previous work by further examining HRQoL and its associated factors among IBD patients with long COVID.

The association between healthcare-access difficulties and lower HRQoL observed in our cohort should also be interpreted in the broader context of COVID-19-related disruption of IBD care. During the pandemic, IBD management was affected by reduced outpatient visits, delayed endoscopic assessment, treatment interruptions or modifications, increased reliance on telemedicine, and patient concerns regarding infection risk. These disruptions may have contributed to impaired disease monitoring, psychological burden, and reduced quality of life among patients with IBD [11].

It is noteworthy that 5-ASA (53.62%) and enteral nutrition (43.83%) were the most commonly reported medications in our cohort, reflecting several features of IBD management in China. Despite 88.09% of participants having CD, for which 5-ASA is not considered standard maintenance therapy in Western guidelines [25, 26], mesalazine continues to be widely prescribed in Chinese clinical practice for mild-to-moderate CD [27, 28]. This pattern is attributable to restricted insurance coverage for biologics, high out-of-pocket costs that limit accessibility, and lower patient acceptance of biologic therapy [29]. Additionally, as the medication question used a multi-select format, many patients likely used 5-ASA in combination with biologics or immunomodulators. This practice is consistent with the Chinese consensus on IBD management and has been reported in other real-world studies from China [30].

Our study identified a significant difference between patients vaccinated with COVID-19 vaccine and those not vaccinated in the development of long COVID in IBD patients. This finding is consistent with previous studies, which suggest that vaccination may reduce long COVID risk by preventing infection, reducing disease severity, or alleviating symptoms in those with long COVID [31]. However, the association between COVID-19 vaccination and long COVID occurrence is different in different studies. This may be related to the different research methods, the time after vaccination and the different definitions of the long COVID. Therefore, it is impossible to draw an exact conclusion on the impact of COVID-19 vaccination on the long COVID [31, 32].

A study published on the *Nature* shows that there is no significant difference between patients vaccinated with COVID-19 vaccine and those not vaccinated with COVID-19 vaccine in the development of long COVID [32], but COVID-19 vaccination has been associated with a lower probability of long COVID in some studies [32, 33]. Previous studies have reported that COVID-19 vaccination was associated with a 15%–40% lower risk of developing long COVID, and the symptoms of long COVID have become better rather than worsened after COVID-19 vaccination [31, 33].

In the present study, 15 of 221 infected IBD patients met the criteria for long COVID, corresponding to a prevalence of 6.79%. This estimate appears numerically lower than some previously reported estimates in broader populations [32]. However, it should be interpreted with caution. Estimates of long COVID prevalence vary substantially across studies, partly because of differences in case definitions, follow-up periods, symptom assessment methods, survey timing, and study populations. Therefore, direct comparison between our cohort and the general population is limited. Given the cross-sectional design of this survey, and the absence of detailed information on factors such as SARS-CoV-2 variant, acute infection severity, vaccination timing, pre-existing symptoms, and IBD disease activity at the time of infection, our study was not designed to determine why the observed prevalence was relatively low. Accordingly, this finding should be considered descriptive and hypothesis-generating rather than explanatory.

Furthermore, we observed that under the normalized management of the COVID-19 pandemic, IBD patients with a pessimistic outlook experienced poorer HRQoL, which is consistent with previous literature [34, 35]. Psychological health issues arising from COVID-19 include anxiety, depression, and post-traumatic stress disorder (PTSD) [35]. Psychosocial factors, together with COVID-19-related experiences such as infection, isolation, physical distancing, incomplete physical recovery, and economic difficulties, may be associated with psychological health problems after COVID-19 infection [35]. In addition, given that IBD patients are more prone to emotional stress and anxiety than the general population, perceived susceptibility to COVID-19 among IBD patients may be associated with greater anxiety and lower HRQoL; however, the cross-sectional design of our study precludes determining the directionality of this association.

Moreover, fatigue is commonly reported among patients with long COVID and may coexist with psychological symptoms such as anxiety and depression [36]. In our study, fatigue was the most commonly reported issue by IBD patients after infected with COVID-19. Fatigue is considered a crucial predictor of HRQoL, and previous reports suggested that fatigue may have a greater impact on overall HRQoL than other symptoms. Early monitoring of fatigue and related symptoms is recommended for survivors of COVID-19 [36]. The observed association between psychological burden and lower HRQoL among IBD patients during the COVID-19 pandemic suggests that greater attention should be paid to the integration of physical and mental health care in this population. It may also suggest the necessity for professional psychological counseling to improve the psychological health in IBD patients under the normalized COVID-19 management [37].

Our research revealed that patients with reduced physical activity have a lower HRQoL. This conclusion aligns with previous literature, indicating that decreased physical activity and difficulties in daily life activities after infected with COVID-19 is associated with poorer HRQoL in the physical health domain [36]. This association may be partly related to protective measures adopted during the COVID-19 pandemic in China and many other countries, including social distancing, cancellation of public gatherings, school closures, quarantine, and lockdowns. These measures may have coincided with reduced physical activity and poorer physical and mental health, although causal relationships cannot be inferred from the present cross-sectional study [38]. Studies also suggest a connection between decreased physical activity and increased risks of anxiety, stress, and exacerbation of chronic conditions, emphasizing the potential therapeutic benefits of physical activity and exercise in mitigating chronic diseases, long COVID, as well as symptoms of depression and anxiety [38].

In our study, only 74 (31.49%) reported never postponing or canceling social activities due to intestinal issues, and only 58 individuals (24.68%) reported that intestinal problems did not affect normal physical activities and leisure. Therefore, the reduced physical activity reported by IBD patients may be related to both gastrointestinal symptom burden and pandemic-related isolation or lockdown measures; however, the cross-sectional design precludes determining the relative contribution or causal direction of these factors. Nevertheless, we still strongly encourage IBD patients to engage in regular moderate physical exercise based on their individual symptoms.

To the best of our knowledge, there are limited reports on the relationship between long COVID infection and COVID-19 vaccination in IBD patients. Additionally, few studies have investigated the HRQoL of IBD patients affected by long COVID. Our research aims to address this gap. Additionally, our study offers valuable guidance for healthcare professionals, dedicated to enhancing the HRQoL of IBD patients under the normalized COVID-19 management.

### Limitations

Our study has several limitations that should be carefully considered when interpreting the findings.

Three inherent limitations are particularly important. First, the cohort was markedly CD-predominant (207 patients with CD vs. 14 with UC), contrary to the usual epidemiologic distribution. The findings therefore predominantly reflect the experience of patients with CD and cannot be confidently generalized to patients with UC or to the broader IBD spectrum. Second, detailed clinical characterization and standardized baseline disease activity measures (e.g., CDAI or Harvey-Bradshaw Index for CD, Mayo score for UC, CRP, fecal calprotectin, or endoscopic assessment) were not collected. Because underlying IBD activity is a major determinant of HRQoL, residual confounding is likely, and the lower HRQoL observed among participants with long COVID cannot be separated from the possible effect of IBD activity. Third, only 15 participants met the long-COVID definition. This modest event count reduced statistical power and precision and limited the robustness of multivariable modeling, particularly for long-COVID-specific estimates. Accordingly, both the prevalence estimate and the observed associations should be considered preliminary.

The number of IBD patients meeting the criteria for long COVID was small (*n* = 15, 6.79%), substantially limiting statistical power. While the multivariate regression demonstrated a statistically significant association between long COVID and reduced HRQoL (*p* = 0.002, B=-8.52), the wide confidence interval (95% CI, -13.85 to -3.18) reflects the limited precision inherent to small event numbers. The prevalence estimate of 6.79% should be considered preliminary, and all conclusions require validation in larger, well-powered cohorts.

Another important limitation is that our study is a cross-sectional survey, which can only identify associations and does not support causal inferences. Although we observed that long COVID was associated with lower HRQoL, we cannot establish the temporal direction or causality of this relationship. Future prospective longitudinal studies are needed.

A further critical limitation is that we did not collect validated disease activity measures (e.g., CDAI, Harvey-Bradshaw Index, Mayo score, CRP, fecal calprotectin, or endoscopic assessment). Disease activity is a well-established primary determinant of HRQoL in IBD, and its absence from our analysis represents a significant potential source of residual confounding. As a remote web-based survey, objective clinical and biochemical indices were not feasible to collect. The SIBDQ bowel domain scores, while reflecting patient-reported symptom burden, do not constitute a formal disease activity assessment. We strongly recommend that future studies incorporate validated disease activity measures to disentangle the effects of IBD activity from those of long COVID on HRQoL. This limitation is particularly relevant because previous observational studies of COVID-19 in patients with IBD have suggested that IBD disease activity may be associated with COVID-19-related outcomes [12].

In addition, our use of a four-week threshold based on the CDC post-COVID conditions framework available at the time of study design and analysis, rather than the WHO definition, may affect the comparability of our prevalence estimate with studies using WHO criteria. Although this broader threshold was chosen given the exploratory nature of our study and the need to identify a wide range of post-COVID-19 symptoms among patients with IBD, it may have influenced the estimated prevalence and the observed associations. Therefore, our findings should be interpreted in the context of the specific case definition used.

Moreover, the marked imbalance between CD (*n* = 207, 88.09%) and UC (*n* = 14, 5.96%) limits the generalizability of our findings to UC-predominant IBD populations. This distribution reflects the referral patterns of our two tertiary centers, which attract a higher proportion of CD patients requiring specialized surgical and medical management.

Furthermore, our web-based survey methodology may introduce selection bias, potentially favoring younger, more educated, and more digitally literate participants, while underrepresenting elderly patients and those without internet access. Additionally, recruitment from only two tertiary referral centers limits geographic and demographic representativeness. We recommend that future studies employ multi-center designs with hybrid online and offline recruitment strategies.

Another potential source of bias is that some aspects of the questionnaire required participants to recall their health status during the COVID-19 pandemic, including the duration and nature of post-COVID-19 symptoms and medication usage, which may introduce recall bias.

Finally, our analysis of factors associated with long COVID development was exploratory, and the small number of long COVID cases precluded robust multivariable modeling for this endpoint.

## Conclusion

In summary, participants meeting the study definition of long COVID reported lower HRQoL than those without long COVID. However, because standardized IBD activity measures were not collected, the observed lower HRQoL cannot be separated from the possible effect of underlying IBD activity and should not be interpreted as an independent or causal effect of long COVID. The cross-sectional design and the small number of long-COVID cases further limit precision and causal inference, and the vaccination finding remains exploratory and unadjusted. These preliminary associations require confirmation in larger prospective studies that incorporate validated clinical, biochemical, and endoscopic measures of IBD activity. Nevertheless, our preliminary observations draw attention to the potential association between long COVID and HRQoL impairment in IBD patients and may encourage further research into this important but understudied area. 

## Supplementary Information

Below is the link to the electronic supplementary material.

Supplementary Material 1

## Data Availability

The datasets used and/or analyzed during the current study are available from the corresponding author on reasonable request.
