# Supplementary material for: Health-related quality of life in IBD patients with long COVID under China’s normalized COVID-19 management
Source: BMC Infect Dis. 2026 Sep 1;26:1728. doi: 10.1186/s12879-026-14323-1 (PMC13621760; doi:10.1186/s12879-026-14323-1)
Supplement: Supplementary file 1 [file 12879_2026_14323_MOESM1_ESM.docx]

**Supplementary Materials**

Table S1. The sequelae of COVID-19[1]

| **Sequelae of COVID-19** | **N=221** |
| --- | --- |
| None  Fatigue  Memory decline and difficulty concentrating  Chest pain, shortness of breath, and wheezing  Decreased sense of smell or taste  Anxiety or depression  Headache or dizziness  Muscle or bone pain  Cough, sore throat, hoarseness  Rashes or sensory abnormalities  Hair loss  Stomach pain and diarrhea  Nausea or vomiting  Fear of cold or fever | 104 (47.06)  76 (34.39)  40 (18.10)  12 (5.43)  11 (4.98)  8 (3.62)  12 (5.43)  12 (5.43)  13 (5.88)  7 (3.17)  23 (10.41)  14 (6.33)  3 (1.36)  9 (4.07) |

Table S2. Pearson correlation analysis

| **Variables** | **r** | **95% CI** | ***p*** |
| --- | --- | --- | --- |
| Gender | 0.056 | -0.076, 0.187 | 0.403 |
| Age | 0.081 | -0.051, 0.211 | 0.228 |
| IBD type | -0.170 | -0.296, -0.039 | **0.011** |
| Operation for IBD | 0.061 | -0.071, 0.192 | 0.365 |
| Complications | 0.194 | 0.064, 0.318 | **0.004** |
| Vaccination | 0.060 | -0.073, 0.190 | 0.376 |
| COVID-19 infection | -0.062 | -0.192, 0.071 | 0.359 |
| Any post-COVID-19 symptoms  Long COVID | 0.370  -0.207 | 0.250, 0.479  -0.330, -0.077 | **＜0.001**  **0.002** |
| Number of COVID-19 infections | -0.166 | -0.292, -0.035 | **0.013** |
| Interval between COVID-19 reinfections | 0.012 | -0.120, 0.144 | 0.862 |
| Difficulty in seeking medical care | -0.262 | -0.381, -0.135 | **＜0.001** |
| Susceptible to COVID-19 | 0.203 | 0.073, 0.326 | **0.002** |
| Unwilling to interaction | 0.202 | 0.072, 0.325 | **0.003** |
| Affecting study or work  Affecting outdoor exercise  Using IBD medicine | 0.220  0.280  0.084 | 0.091, 0.342  0.154, 0.397  -0.048, -0.214 | **＜0.001**  **＜0.001**  0.211 |
|  |  |  |  |

**English language version of the questionnaire**

This is a web-based survey, and we used Wenjuanxing (www.wjx.cn) to create the questionnaire.

**Section 1: Demographic Information**

**Q1:** What is your gender?
□ Male
□ Female

**Q2:** What is your age?
□ ≤16 years
□ 17-40 years
□ >40 years

**Q3:** What is your place of residence?
Province: ____ City: ____ (Participants to fill in based on their actual situation)

**Q4:** What is your highest level of education?
□ Below high school
□ High school graduate
□ University
□ Postgraduate

**Q5:** What was your age at the time of IBD diagnosis?
□ ≤16 years
□ 17-40 years
□ >40 years

**Q6:** What is your diagnosis? Crohn's Disease (CD) / Ulcerative Colitis (UC)?
□ CD
□ UC
□ Unclear type

**Q7:** Have you undergone surgery for IBD in the past?
□ Yes
□ No

**Q8:** What medications are you currently using? (Multiple choice)
□ None
□ Enteral nutrition
□ Parenteral nutrition
□ 5-ASA (Mesalazine)
□ Corticosteroids
□ Immunosuppressants (Azathioprine, Methotrexate)
□ Adalimumab
□ Infliximab
□ Ustekinumab
□ Vedolizumab

**Q9:** Do you have any other comorbidities? (Multiple choice)
□ None
□ Hypertension
□ Diabetes
□ Kidney disease
□ Tuberculosis
□ Liver disease
□ Other autoimmune diseases
□ Obesity/Overweight
□ Other

**Section 2: COVID-19 Related Questions**

**Q10:** What is your COVID-19 vaccination status?
□ Not vaccinated
□ Completed the first dose
□ Completed the second dose
□ Completed the third dose
□ Completed the fourth dose

**Q11:** Have you ever been infected with COVID-19?
□ Not infected
□ Infected, recovered
□ Infected, currently infected
***The following questions are for participants who have been infected with COVID-19:**

**Q12:** What symptoms did you experience after being infected with COVID-19? (Multiple choice)
□ Fever
□ Dry cough
□ Fatigue
□ Loss of smell
□ Loss of taste
□ Nasal congestion or runny nose
□ Sore throat
□ Conjunctivitis
□ Muscle pain
□ Diarrhea
□ Asymptomatic

**Q13:** How long did your COVID-19 symptoms last?

□< 4 weeks

□**≥** 4 weeks

**Q14:** How many times have you been infected with COVID-19?

□Once

□Twice

□Three times

□More than three times

**Q15:** What was the interval between your COVID-19 infections?

□< 1 month

□1 - 3 months

□4 - 6 months

□> 6 months

**Q16:** What post-recovery symptoms did you experience after recovering from COVID-19? (Multiple choice) [1]

□None

□Fatigue

□Memory loss, difficulty concentrating

□Chest pain, shortness of breath

□Loss of smell or taste

□Anxiety or depression

□Headaches or dizziness

□Muscle or skeletal pain

□Cough, sore throat, hoarseness

□Rash or sensory abnormalities

□Hair loss

□Stomach pain, diarrhea

□Nausea or vomiting

□Chills or fever
**Q17:** What treatments did you receive after being infected with COVID-19? (Multiple choice)

□Antipyretics (ibuprofen, acetaminophen, etc.)

□Steroids (hydrocortisone, dexamethasone, prednisone, budesonide, etc.)

□Antiviral drugs (oseltamivir, ribavirin, amantadine, etc.)

□Traditional Chinese medicine (Lianhua Qingwen, Pudi Lan, Huo Xiang Zheng Qi, etc.)

□Baricitinib

□Tocilizumab

□No medication

**Section 3: Short Inflammatory Bowel Disease Questionnaire (SIBDQ)** [2-4]

**Q18:** How often has the feeling of fatigue or of being tired and worn out been a problem for you during the last 2 weeks?
□ All of the time
□ Most of the time
□ A good bit of the time
□ Some of the time
□ A little of the time
□ Hardly any of the time
□ None of the time

**Q19:** How often during the last 2 weeks have you had to delay or cancel a social engagement because of your bowel problem?
□ All of the time
□ Most of the time
□ A good bit of the time
□ Some of the time
□ A little of the time
□ Hardly any of the time
□ None of the time

**Q20:** How much difficulty have you had, as a result of your bowel problems, doing leisure or sports activities you would have liked to have done during the last 2 weeks?
□ A great deal of difficulty, activities made impossible
□ A lot of difficulty
□ A fair bit of difficulty
□ A little difficulty
□ Hardly any difficulty
□ No difficulty
□ The bowel problems did not limit sports or leisure activities

**Q21:** How often during the last 2 weeks have you been troubled by pain in the abdomen?
□ All of the time
□ Most of the time
□ A good bit of the time
□ Some of the time
□ A little of the time
□ Hardly any of the time
□ None of the time

**Q22:** How often during the last 2 weeks have you felt depressed or discouraged?
□ All of the time
□ Most of the time
□ A good bit of the time
□ Some of the time
□ A little of the time
□Hardly any of the time
□ None of the time

**Q23:** Overall, in the last 2 weeks, how much of a problem have you had with passing large amounts of gas?
□ A major problem
□ A big problem
□ A significant problem
□ Some trouble
□ A little trouble
□ Hardly any trouble
□ No trouble

**Q24:** Overall, in the last 2 weeks, how much of a problem have you had maintaining or getting to the weight you would like to be?
□ A major problem
□ A big problem
□ A significant problem
□ Some trouble
□ A little trouble
□ Hardly any trouble
□ No trouble

**Q25:** How often during the last 2 weeks have you felt relaxed and free of tension?
□ All of the time
□ Most of the time
□ A good bit of the time
□ Some of the time
□ A little of the time
□ Hardly any of the time
□ None of the time

**Q26:** How much of the time during the last 2 weeks have you been troubled by a feeling of having to go to the bathroom even though your bowels were empty?
□ All of the time
□ Most of the time
□ A good bit of the time
□ Some of the time
□ A little of the time
□ Hardly any of the time
□ None of the time

**Q27:** How much of the time during the last 2 weeks have you felt angry as a result of your bowel problem?
□ All of the time
□ Most of the time
□ A good bit of the time
□ Some of the time
□A little of the time
□ Hardly any of the time
□ None of the time

**Section 4: Questions Related to Physical and Mental Health in IBD Patients**

**Q28:** What was your experience with seeking medical care for IBD in the past month? (Multiple choice)
□ No need to visit a medical facility
□ No difficulty in seeking medical care/similar to previous experiences
□ Difficulty obtaining medication
□ Difficulty scheduling appointments
□ Difficulty scheduling endoscopy or other imaging tests
□ Difficulty getting hospitalized
□Other

**Q29:** Do you feel that you are more susceptible to COVID-19 compared to the general population?
□ Strongly agree
□ Agree
□ Neither agree nor disagree
□ Disagree
□ Strongly disagree

**Q30:** Do you avoid contact with others due to fear of contracting COVID-19?
□ Strongly agree
□ Agree
□ Neither agree nor disagree
□ Disagree
□ Strongly disagree

**Q31:** In the past month, has your study/work/income been negatively affected by COVID-19?
□ Strongly agree
□ Agree
□ Neither agree nor disagree
□ Disagree
□ Strongly disagree

**Q32:** In the past month, have your outdoor activities/exercise decreased compared to before?
□ Strongly agree
□ Agree
□Neither agree nor disagree
□ Disagree
□ Strongly disagree

**Q33:** Have you been taking IBD medication regularly in the past month?
□ Stopped taking medication
□ Irregular usage occasionally
□ Frequent irregular usage
□ Taking medication as prescribed by the doctor

**Q34:** Have you participated in the "The impact of COVID-19 on the HRQoL of IBD patients after the end of the zero-COVID policy"[5]?

□Yes

□No

***References***

1.	Thaweethai T, Jolley SE, Karlson EW, Levitan EB, Levy B, McComsey GA, McCorkell L, Nadkarni GN, Parthasarathy S, Singh U *et al*: **Development of a Definition of Postacute Sequelae of SARS-CoV-2 Infection**. *Jama* 2023, **329**(22):1934-1946.

2.	de Bock E, Filipe MD, Meij V, Oldenburg B, van Schaik FDM, Bastian OW, Fidder HF, Vriens MR, Richir MC: **Quality of life in patients with IBD during the COVID-19 pandemic in the Netherlands**. *BMJ Open Gastroenterol* 2021, **8**(1).

3.	Roseira J, Sousa HT, Marreiros A, Contente LF, Magro F: **Short Inflammatory Bowel Disease Questionnaire: translation and validation to the Portuguese language**. *Health Qual Life Outcomes* 2021, **19**(1):59.

4.	Liu R, Tang A, Wang X, Shen S: **Assessment of Quality of Life in Chinese Patients With Inflammatory Bowel Disease and their Caregivers**. *Inflamm Bowel Dis* 2018, **24**(9):2039-2047.

5.	Wang R, Liu P, Fan C, Liu J, Jiang H, Ren J, Zhao Y, Zheng T: **The impact of the COVID-19 surge after the end of China's Zero-COVID policy on the health-related quality of life of IBD patients**. *BMC Gastroenterol* 2024, **24**(1):312.

Figure S1. COVID-19 related questions (Comparison between three or more groups). (a) Number of COVID-19 infections; (b) Susceptible to COVID-19; (c) Unwilling to interaction; (d) Affecting study or work; (e) Affecting outdoor exercise (* p< 0.05; ** p< 0.01; *** p< 0.001; **** p< 0.0001).
